# A Novel Approach - The Propensity to Propagate (PTP) Method for Controlling for Host Factors in Studying the Transmission of Mycobacterium Tuberculosis

**DOI:** 10.1371/journal.pone.0097816

**Published:** 2014-05-21

**Authors:** Hanna Nebenzahl-Guimaraes, Martien W. Borgdorff, Megan B. Murray, Dick van Soolingen

**Affiliations:** 1 National Institute for Public Health and the Environment (RIVM), Bilthoven, The Netherlands; 2 Life and Health Sciences Research Institute (ICVS), School of Health Sciences, University of Minho, Braga, Portugal; 3 ICVS/3B's, PT Government Associate Laboratory, Braga/Guimarães, Portugal; 4 Public Health Service, Amsterdam, Netherlands; 5 Department of Clinical Epidemiology, Academic Medical Centre, University of Amsterdam, Amsterdam, Netherlands; 6 Department of Global Health and Social Medicine, Harvard Medical School, Boston, United States of America; 7 Department of Medical Microbiology, Radboud University Nijmegen Medical Centre, Nijmegen, The Netherlands; Institut de Pharmacologie et de Biologie Structurale, France

## Abstract

**Rationale:**

Understanding the genetic variations among Mycobacterium tuberculosis (MTB) strains with differential ability to transmit would be a major step forward in preventing transmission.

**Objectives:**

To describe a method to extend conventional proxy measures of transmissibility by adjusting for patient-related factors, thus strengthening the causal association found with bacterial factors.

**Methods:**

Clinical, demographic and molecular fingerprinting data were obtained during routine surveillance of verified MTB cases reported in the Netherlands between 1993 and 2011, and the phylogenetic lineages of the isolates were inferred. Odds ratios for host risk factors for clustering were used to obtain a measure of each patient's and cluster's propensity to propagate (CPP). Mean and median cluster sizes across different categories of CPP were compared amongst four different phylogenetic lineages.

**Results:**

Both mean and median cluster size grew with increasing CPP category. On average, CPP values from Euro-American lineage strains were higher than Beijing and EAI strains. There were no significant differences between the mean and median cluster sizes among the four phylogenetic lineages within each CPP category.

**Conclusions:**

Our finding that the distribution of CPP scores was unequal across four different phylogenetic lineages supports the notion that host-related factors should be controlled for to attain comparability in measuring the different phylogenetic lineages' ability to propagate. Although Euro-American strains were more likely to be in clusters in an unadjusted analysis, no significant differences among the four lineages persisted after we controlled for host factors.

## Introduction

Transmission of *Mycobacterium tuberculosis* (MTB) occurs through aerosol droplets. Subsequent cases in transmission chains result in “clusters” of patients who share Mtb strains of the same genotype or molecular fingerprint [1]. Cluster sizes vary widely, which may reflect the fact that strains do not spread equally or that they differ in their rate of progression to active TB disease. The identification of strains that cause large tuberculosis (TB) outbreaks, such as CDC1551 or Harlingen [2], [3], has lead to studies on the virulence of such strains. Indeed molecular epidemiologic studies have suggested that some strains are more successfully transmitted than others [4]–[6]. The mechanisms however governing this variability remain largely unknown, with much research focused on the contribution of host risk factors. In the Netherlands for example, age, sex, homelessness, alcohol or drug abuse, living in an urban area and smear positivity have all been associated to increased transmissibility [7]. There is substantial evidence however to suggest that bacterial factors also contribute to variability in cluster size and the extent of transmission of TB. For example, Verhagen and colleagues showed that newly diagnosed index cases in a larger cluster infected more people than did newly diagnosed cases in smaller clusters [8]. This implies that clusters not only grow over time because of well-known patient risk factors for TB transmission, but also because the strain itself generates an increased number of tuberculin skin test-positive contacts, and spreads more effectively than other strains.

Phylogenetic lineages reflect evolutionary divergence associated with different geographical regions [9]. Beijing lineage strains, for example, are predominantly found in Asia, yet are widely disseminated and present in more countries than any other lineage strain. This suggests that this evolutionary lineage may have evolved unique properties leading to its successful clonal expansion [10], [11]. To date, studies examining the association between phylogenetic lineages of MTB and transmissibility have typically used DNA fingerprinting clustering rates as measures of transmissibility, with very few adjusting for host-related factors.

Since preventing transmission of MTB is key to a sustained decline in TB incidence, understanding the genetic variations between strains with differential ability to transmit would be a major step forward. In order to distinguish bacterial factors associated with transmission from those that pertain to the host however, the influence of host-related factors needs to be addressed. In the Netherlands, a nationwide surveillance of TB including structural DNA fingerprinting of all *M. tuberculosis* isolates has been in place since 1993. Patient information is available for all registered TB cases, of which there are approximately one thousand per year. Here, we describe a method to complement and extend the conventional use of cluster size and proportion of cases in a cluster as proxy measures of transmissibility by adjusting for patient related factors, thus strengthening the causal association found with bacterial factors. Since cluster size may reflect both the propensity of a strain to be transmitted and to cause disease given an infection, we have chosen to use the term “propagation” instead of transmissibility as a more accurate description of cluster growth.

## Methods

### Data Collection and DNA Fingerprinting

The National Institute for Public Health and the Environment (RIVM) in Bilthoven, The Netherlands, serves as a reference laboratory for the secondary laboratory diagnosis of all TB cases in The Netherlands, offering identification, drug susceptibility testing, and molecular typing. DNA fingerprints of all nationwide MTB complex isolates and their cluster status have been stored in a RFLP database since 1993. The Registration Committee of the Netherlands Tuberculosis Register (NTR) approved this retrospective study and provided demographic and clinical information for patients. Because these data are de-identified by name, DNA fingerprinting results from the RIVM were linked on the basis of sex, date of birth, year of diagnosis and postal code. All notified MTB culture-positive cases between 1993 and 2011 were included in the study. In case of patients with multiple isolates, only the isolate with the earliest date of diagnosis was included. Contaminated isolates were excluded from the database.

Isolates recovered from patients between 1993 and 2009 underwent IS*6110* and polymorphic GC-rich sequence (PGRS) restriction fragment length polymorphism (RFLP) typing (n = 15,073), and those from 2004 onward to variable number of tandem repeat (VNTR) typing (n = 5,870) [12], [13], In the period of 2004–2008 both RFLP and VNTR typing were performed [14]. In addition, 4,433 randomly selected isolates were spoligotyped. We defined a cluster as groups of patients who shared TB isolates with identical RFLP or VNTR patterns or, if strains had fewer than five IS*6110* copies, identical PGRS RFLP patterns [15].

### Classification into phylogenetic lineages

The phylogenetic label of a spoligotyped isolate was used to infer the lineage of isolates belonging to the same RFLP or VNTR cluster as the spoligotyped isolate. Following this, the MIRU-VNTRplus online tool was used to perform MIRU Best Match Analysis (stringent cut-off of 0·17) followed by MIRU Tree-based identification to identify the phylogenetic lineages of strains with MIRU patterns [16]. Resulting matched phylogenetic lineages from clustered isolates were extrapolated to the remaining isolates of the respective clusters. Remaining strains without an inferred lineage were assigned one on the basis of RFLP similarity (≥80%) to a reference dataset of pre-identified strains with RFLP patterns in a tree generated by the neighbor-joining method with the Kimura 2 parameter on BioNumerics software for Windows (version 6·6, Applied Maths). The same procedure was repeated for strains with RFLP PGRS patterns. Finally, any remaining MIRU-typed strains without an inferred lineage were subjected to MIRU Best Match Analysis (relaxed cut-off of 0·3). This was purposely left as the last in the series of steps for the classification of lineages as it is the least optimized for minimizing fine-tuned mismatching that can occur as an exception among strains belonging to the Euro-American family [17].

**Figure 1:**
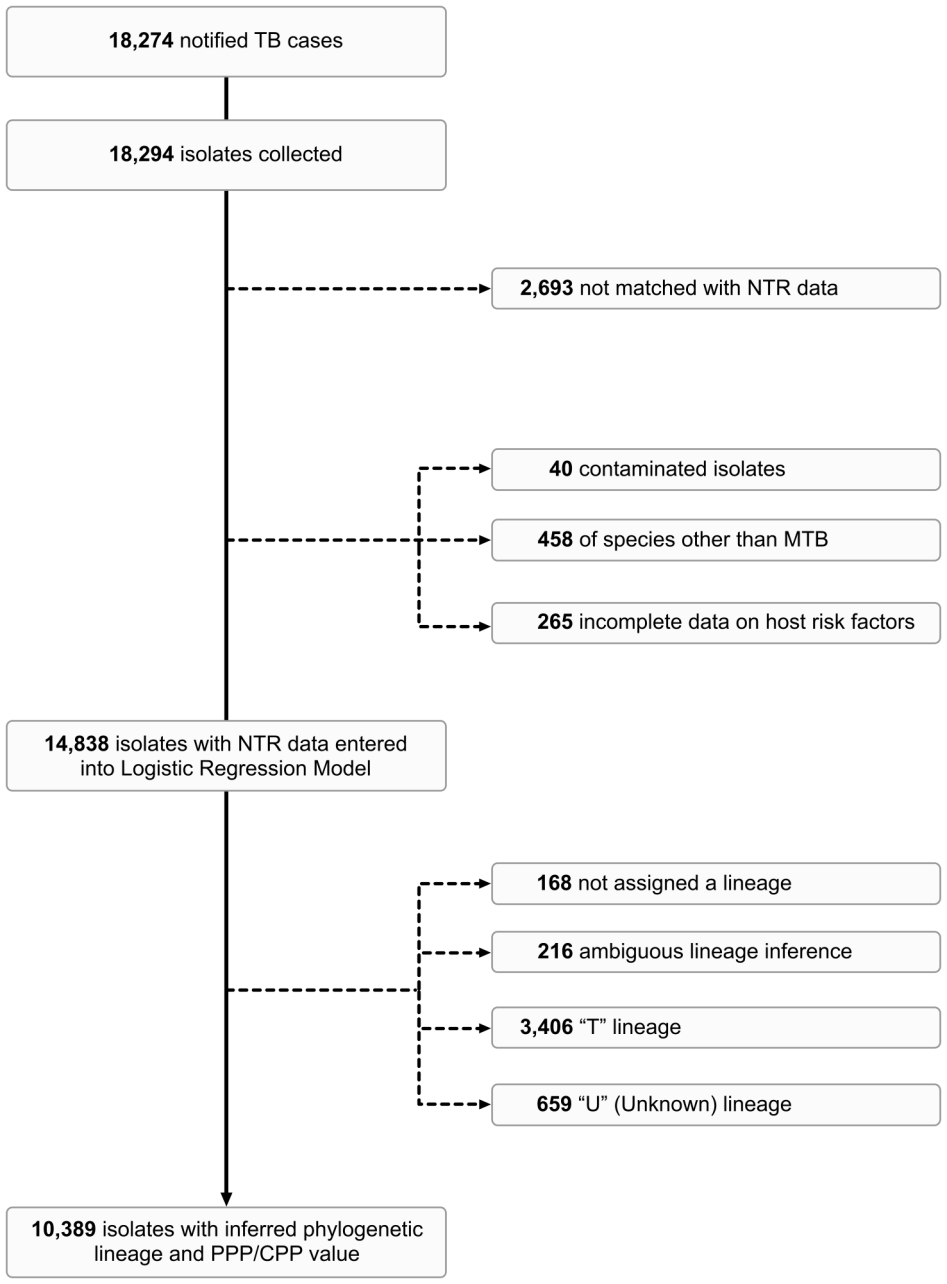
Flow-diagram of exclusion criteria applied to dataset.

Four major phylogenetic lineages were identified: Euro-American, Central Asian Strain (CAS), East-African-Indian (EAI) and Beijing (Table S1). Strains not assigned a phylogenetic lineage or assigned more than one major phylogenetic family per cluster were excluded from analysis. Strains classified as either “T” or “U” (Unknown) were also excluded due to the ambiguity of these classifications (Figure 1).

We considered the possibility that the use of spoligotyping, MIRU- or RFLP-typing for inferring phylogenetic lineages in this study may have resulted in misclassification of lineage, due to the propensity of these markers for convergent evolution and resulting homoplasies [18]. To assess this, we compared the inferred phylogenetic lineages with those determined using single nucleotide polymorphisms (SNP) markers in a subset of strains (n = 248) that were also whole-genome sequenced [19].

### Statistical Analysis

We used a logistic regression model to determine independent host risk factors including demographic, behavioral, and sputum smear status, for clustering. Variables with p-values <0·20 were entered into a multivariate model. Crude and adjusted odds ratios (OR) are presented with 95% confidence intervals (CI). Estimates for the adjusted ORs were each multiplied as weights to calculate each patient's propensity to propagate (PPP). The geometric mean of PPP values belonging to a cluster was taken as the overall measure of a cluster's propensity to propagate (CPP). Confidence intervals for the median CPP by phylogenetic lineage were calculated using nonparametric bootstrapping methods based on 10,000 replicates. An analysis of variance (ANOVA) with Bonferroni correction was performed to determine CPP comparability among the four phylogenetic lineages. We repeated this step on a validation subset of strains (n = 2,136) whose lineages were determined using the highly reliable MIRU Best Match Analysis (stringent cut-off) and SNP markers [9]. We also explored the variability of CPP by phylogenetic lineage stratified by host region of origin, by repeating the ANOVA on a subset of clusters composed of patients of a particular region only (Europe versus Asia). In a sensitivity analysis, we checked the consequences of excluding extra-pulmonary cases from the dataset. Finally, the proportion of clustered isolates was calculated for each phylogenetic lineage. Mean and median cluster size (plus interquartile ranges and 95% CI, respectively) were calculated for three increasing CPP categories (<0·5, 0·5–0·8 and >0·8) for each of the four phylogenetic lineages. SAS software for Windows, version 9·3, was used for statistical analyses.

## Results

During the period January 1993 to December 2011, 18,294 isolates were collected from 18,274 notified TB cases in the Netherlands and their clustering status ascertained, of which 15,601 (85%) were successfully matched with the NTR data. Of these, 14,838 (94%) were non-contaminated MTB cultures with completely ascertained information on host risk factors (Figure 1). The mean age of MTB positive TB cases was 41 years (SD, 20); 8,859 (60%) were male; and 10,005 (67%) were foreign-born.

### Host-related factors for clustering

**Figure 2:**
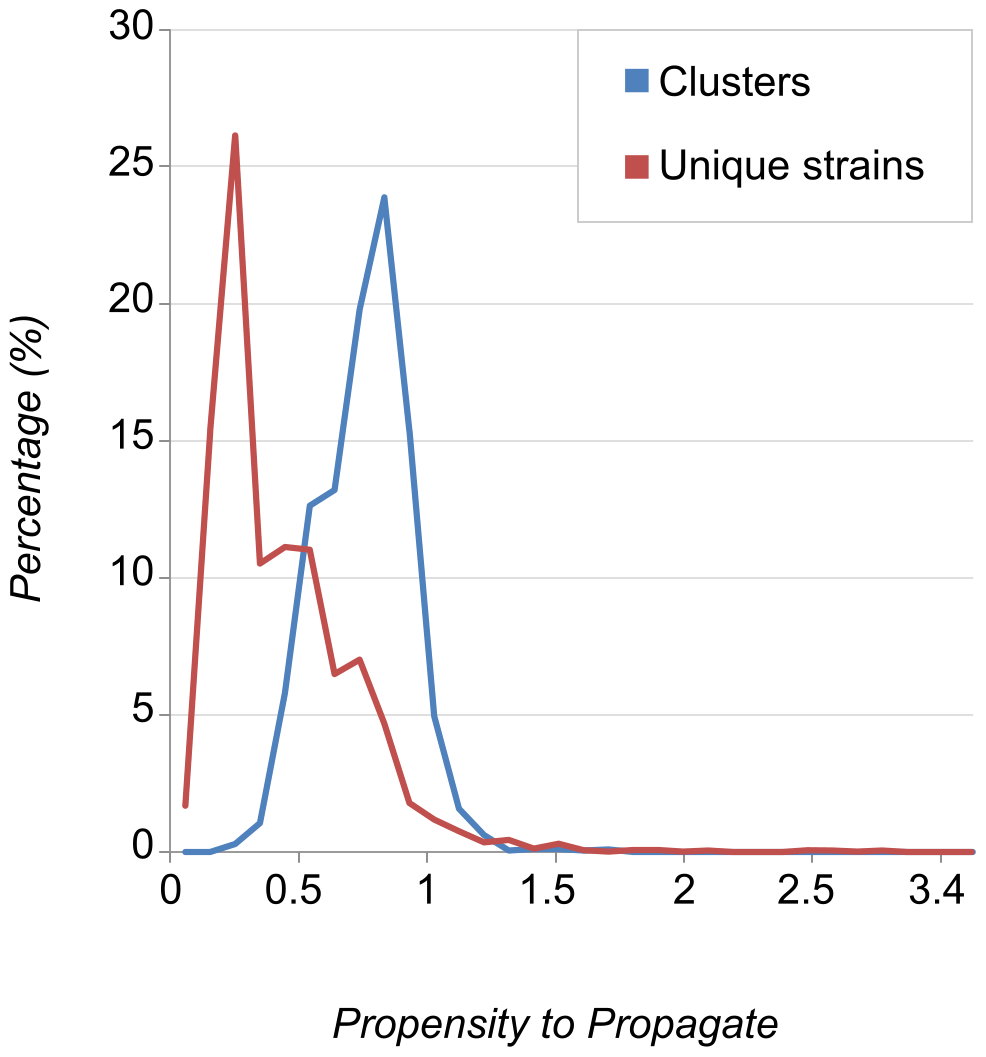
Distribution of Propensity to Propagate values.

Of the 14,838 strains with both DNA fingerprinting and host-related data, 8,585 were clustered (57·9%) and 6,253 were non-clustered (42·1%). Table 1 shows that patients were more likely to be in a cluster if they were smear-positive, had a pulmonary manifestation and were younger, male, alcohol or IV drug users, homeless, a health-care worker, native Dutch or foreign-born from Africa or the Americas. Patients were less likely to be in a cluster if they had travelled to an endemic area in the past two months or were foreign-born from Asia or Europe. In the multivariate model, all risk factors for clustering remained significant with the exception of alcohol consumption, being a health-care worker or being a foreign-born from the Americas. Being a foreign-born from Africa turned into a protective factor against clustering after adjustment in multivariate analysis. Resulting values for PPP and CPP ranged from 0 (a low risk profile for clustering, i.e. an elderly female patient with extra-pulmonary, smear-negative TB and no behavioral risk factors) to 3·9 (a high risk profile i.e. a young (<30 years) male patient with pulmonary, smear-positive TB and at least one behavioral risk factor), with the distribution of CPP values skewed to the right of PPP values from patients with unique isolates (Figure 2).

**Table 1:** Host risk factors for clustering of MTB in the Netherlands, 1993–2011.

		No. (%) in clustering state:			
Category and case group		Clustered	Non-clustered	OR (95% CI)	Adjusted OR (95% CI)
**Sex**	Males	5385 (60.8)	3474 (39.2)	1 (Ref)	1 (Ref)
	Females	3200 (53.5)	2779 (46.5)	**0.74 (0.70–0.79)**	**0.87 (0.81–0.93)**
					
**Age at diagnosis (years)**	0–15	366 (69.2)	163 (30.8)	**1.28 (1.11–1.56)**	1.05 (0.86–1.29)
	16–30	3383 (63.7)	1929 (36.3)	1 (Ref)	1 (Ref)
	31–45	2485 (60.9)	1593 (39.1)	**0.89 (0.82–0.97)**	**0.86 (0.78–0.94)**
	46–60	1254 (59.5)	852 (40.5)	**0.84 (0.76–0.93)**	**0.77 (0.69–0.86)**
	61–75	715 (45.1)	871 (54.9)	**0.47 (0.42–0.52)**	**0.40 (0.35–0.45)**
	76–90	370 (31.6)	800 (68.4)	**0.26 (0.23–0.30)**	**0.19 (0.17–0.23)**
	>90	12 (21.1)	45 (78.9)	**0.15 (0.08–0.29)**	**0.12 (0.06–0.22)**
					
**Disease Classification**	Pulmonary	5107 (61.6)	3187 (38.4)	1 (Ref)	1 (Ref)
	Extrapulmonary	2465 (51.2)	2347 (48.8)	**0.66 (0.61–0.70)**	**0.76 (0.69–0.83)**
	Pulmonary-extrapulmonary	1013 (58.5)	719 (41.5)	**0.88 (0.79–0.98)**	**0.90 (0.80–1.01)**
					
**Smear-positivity**	No	5068 (54.7)	4205 (45.3)	1 (Ref)	1 (Ref)
	Yes	3517 (63.2)	2048 (36.8)	**1.43 (1.33–1.53)**	**1.17 (1.07–1.27)**
					
**Alcohol consumption**	No	8426 (57.6)	6200 (42.4)	1 (Ref)	1 (Ref)
	Yes	159 (75.0)	53 (25.0)	**2.20 (1.61–3.01)**	1.29 (0.92–1.80)
					
**Drug-use**	No	8213 (57.0)	6193 (43.0)	1 (Ref)	1 (Ref)
	Yes	372 (86.1)	60 (13.9)	**4.67(3.55–6.15)**	**2.75 (2.05–3.67)**
					
**Homelessness**	No	8362 (57.4)	6198 (42.6)	1 (Ref)	1 (Ref)
	Yes	223 (80.2)	55 (19.8)	**3.0 (2.23–4.04)**	**1.58 (1.15–2.18)**
					
**Health-care worker**	No	8463 (57.8)	6187 (42.2)	1 (Ref)	1 (Ref)
	Yes	122 (64.9)	66 (35.1)	**1.35 (1.00–1.83)**	**1.00 (0.73–1.38)**
					
**Traveler to endemic areas**	No	8423 (58.0)	6090 (42.0)	1 (Ref)	1 (Ref)
	Yes	162 (49.8)	163 (50.2)	**0.72 (0.58–0.90)**	**0.58 (0.46–0.73)**
					
**Origin**	Native Dutch	2343 (58.4)	1667 (41.6)	1 (Ref)	1 (Ref)
	Foreign-born (Asia)	1247 (39.0)	1949 (61.0)	**0.41 (0.37–0.45)**	**0.28 (0.25–0.31)**
	Foreign-born (Africa)	3253 (65.0)	1749 (35.0)	**1.18 (1.09–1.29)**	**0.76 (0.69–0.84)**
	Foreign-born (America)	704 (72.1)	273 (27.9)	**1.64 (1.41–1.91)**	1.06 (0.90–1.25)
	Foreign-born (Europe)	415 (53.1)	366 (46.9)	**0.72 (0.62–0.84)**	**0.43 (0.37–0.51)**
**Total cases**		8585 (57.9)	6253 (42.1)		

OR, odds ratio; CI, confidence interval; Statistically significant OR are highlighted in bold.

### Host-related factors by phylogenetic lineage

**Figure 3:**
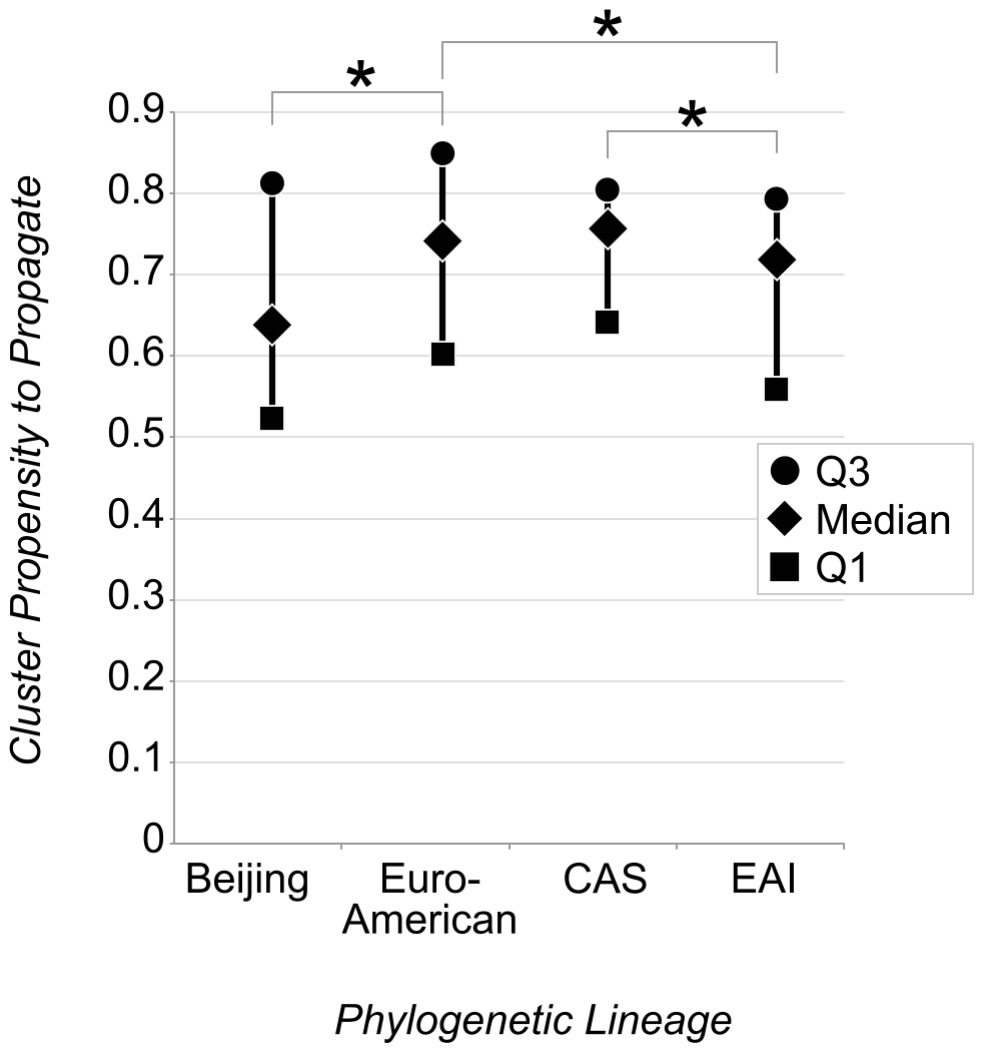
Distribution of Cluster Propensity to Propagate by 4 Phylogenetic Lineages. * 0.05 Level of Significance. Q1 – Lower Quartile; Q3 – Upper Quartile.

Of the 10,389 *M. tuberculosis* isolates which had both a CPP and/or PPP value and an assigned phylogenetic lineage, 6,595 were classified as Euro-American (63·5%), 1,327 as CAS (12·8%), 1,422 as EAI (13·7%) and 1,045 as Beijing (10·0%). The excluded 15% of strains that were not matched with the NTR data fall into a similar lineage distribution. Lineage misclassification was estimated at 19%, with 200 out of 248 strains in this study having concordant lineage classifications to SNP-based inferences. Of the 10,389 strains, 4,491 (43·2%) were non-clustered and the remainder consisted of 1,505 clusters, representing 175 CAS clusters, 972 Euro-American, 202 EAI and 156 Beijing. Median values for CPP were 0·64 (95% CI: 0·57–0·67), 0·76 (95% CI: 0·73–0·77), 0·75 (95% CI: 0·71–0·76) and 0·72 (95% CI: 0·70–0·73) for Beijing, Euro-American, CAS and EAI strains. CPP values from strains of the Euro-American lineage were on average higher than those of Beijing and EAI strains, and CAS strains were also on average higher than EAI strains at a 0·05 level of significance (Figure 3). CPP values of strains belonging to the validation subset of strains classified using high reliability markers showed a similar trend, with the median CPP of strains of the Euro-American lineage remaining on average higher than those of Beijing and EAI strains at a 0·05 level of significance. Repeating the ANOVA on clusters composed of patients of European origin only (n = 277) showed a significantly lower mean CPP in clusters of the Euro-American strain (0·73; 95% CI: 0·70–0·76) compared to that of in clusters of Beijing (0·89; 95% CI: 0·80–0·98) or CAS (0·86; 95% CI: 0·77–0·96) strains, at a 0·05 level of significance. In the subset of clusters composed of patients of Asian origin only (n = 57), mean CPP values were 0·46 (95% CI: 0·43–0·49), 0.40 (95% CI: 0·32–0·48), 0·40 and 0·43 (95% CI: 0·39–0·47), for Beijing, Euro-American, CAS (n = 1) and EAI strains, respectively. Excluding extra-pulmonary cases (n = 4,812) from the dataset and logistic regression model resulted in Beijing strains maintaining the lowest median CPP (0·71, 95% CI: 0·65–0·78) compared to that of Euro-American (0·85, 95% CI: 0·82–0·85), CAS (0·84, 95% CI: 0·80–0·85) and EAI (0·83, 95% CI: 0·75–0·86) strains.

### Propagation by phylogenetic lineage

**Figure 4:**
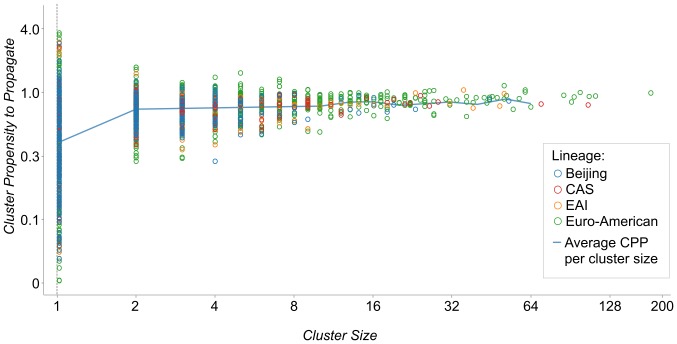
Distribution of Propensity to Propagate by Cluster Size.

**Figure 5:**
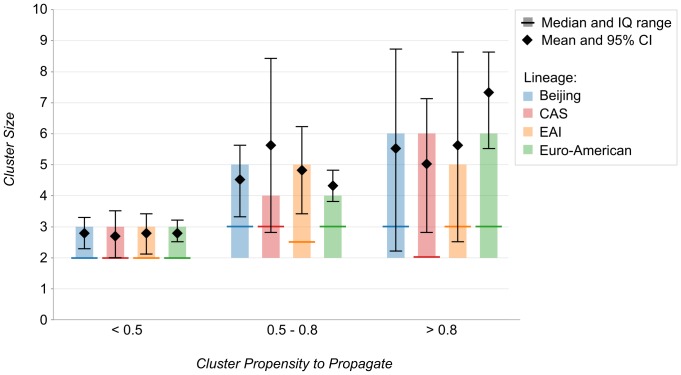
Distribution of Cluster Propensity to Propagate by Cluster Size and 4 Phylogenetic Lineages.

The proportion of clustered isolates was 60·7% (95% CI, 59·5–61·9) for Euro-American strains, 49·2% (95% CI, 46·5–51·9) for CAS strains, 51·1% (95% CI, 48·5–53·7) for EAI strains and 49·4% (95% CI, 46·4–52·4) for Beijing strains. Both minimum and average PPP/CPP per cluster size increased with rising cluster size (Figure 4). Likewise, mean and median cluster size grew with increasing CPP category (Figure 5). There were no significant differences between the mean and median values of cluster size between the four phylogenetic lineages within each CPP category.

## Discussion

In this long-term Netherlands-based study, we compared the propensity to propagate of four major MTB lineages using a novel method designed to differentiate host and bacterial factors associated with strain transmissibility and progression. We found that although Euro-American strains were more likely to be found in clusters in an unadjusted analysis, no significant differences among the four different lineages persisted after we controlled for host factors.

The range of host factors associated with clustering that we identified in this study include demographic (age, gender and geographic origin), clinical (pulmonary manifestation and smear-positivity) and behavioral (drug-use, homelessness) determinants that have been identified in previous studies in this (and other) settings [7], [20]. The clearly skewed distribution of cluster CPP values to the right of PPP values from patients with unique isolates confirms the role of host-related factors in propagation. The method described in this study to correct for host-related factors in transmission enables the identification of highly propagating strains (i.e. belonging to a larger than average cluster size for its CPP score) from non-propagating ones (i.e. non-clustered isolates with a high PPP). This selection process is useful to hone in on a crisp phenotype that is necessary to study bacterial factors associated with transmission, by means of genomic comparison in future whole-genome sequencing studies [21]. It would for example be interesting to subject the CDC1551 outbreak to our new approach in order to separate host risk factors from the true bacteriological component.

Our finding that the distribution of Cluster Propensity to Propagate scores was not equal across the Euro-American, Beijing, CAS and EAI lineages supports the notion that host-related factors need to be controlled for in order to attain comparability in measuring the ability of different phylogenetic lineages to propagate. Other previous studies in low prevalence settings such as Montreal and San Francisco have found the EAI lineage to be associated with lower rates of transmission [22], and the Euro-American lineage three times more likely to cause a secondary case [23]. The former adjusted their OR for clustering for age, whilst the rate measure used in the latter did not adjust for host-related factors. Discrepancies between results from studies measuring transmissibility between phylogenetic lineages may therefore partly be due to differences in how and if host-related factors are controlled for at all. This also seems important in the light of studies on co-evolution between bacteria and hosts; to facilitate a meaningful interpretation such studies should take patient risk factors for transmission and breakdown to disease into consideration [24]. In high prevalence settings this may be especially challenging.

A major strength of our study was the use of a large sample size over a long time period to accurately quantify the contribution of host-related factors in clustering within this setting. With 69% of patients being foreign-born from 159 different countries, our study sample is also globally representative; given the phylogeographic diversity of the major MTB lineages this is crucial to perform comparative analyses to identify associations between strain lineages and transmissibility. There is also an advantage in conducting this analysis in a low prevalence setting such as the Netherlands where the majority of people are susceptible and not vaccinated with BCG. This means that cluster sizes more closely reflect the biological underpinning of increased transmissibility rather than the proportion of the population that is still susceptible to MTB. Finally, our use of mean and median cluster size (therefore excluding non-clustered strains) across CPP categories instead of clustering rates decreases possible bias from the over-representation of foreign-born patients, associated with non-clustered strains from reactivation of latent TB infections acquired before immigration, among non-Euro-American strains (74·3%, 95% CI: 73·0–75·6) versus Euro-American strains (53·3%, 95% CI: 52·2–54·4).

Although our results contrast with those from studies carried out in other populations where Beijing has been associated with greater virulence and transmissibility [25]–[27], they are consistent with those from other low incidence immigrant-receiving settings such as the United States and Canada where it was concluded that Beijing strains do not pose more of a public health threat than non-Beijing strains [23], [28]. The successful spread of this genotype in Asia and other parts of the world may therefore be related to a higher ability to withstand exposure to antituberculosis drugs and BCG vaccination, rather than a higher ability to propagate [11], [29].

It has also been hypothesized that lineages that are rare in a specific human population are not adapted to transmit and cause secondary cases [23]. In Sweden for example, despite the close proximity to Russia and the Baltic states, Beijing was found to have a lower clustering rate, no absolute increase in number over time and very little observed transmission from immigrants to indigenous population [30]. In our study, there was no statistically significant difference between the median and mean cluster sizes of Beijing versus Euro-American strains after taking host propensity to propagate factors into account. This was also found to be the case for EAI and CAS strains, which suggests that imported strains in the Netherlands are not necessarily less adapted to the native host population and are just as likely to propagate as locally occurring strains of the Euro-American lineage. A lower mean CPP of Euro-American versus non-Euro-American strains found in clusters of European origin only suggests the possibility of co-evolution between phylogenetic lineages to their sympatric host population, as has been previously reported [23]. No significant differences were found however between CPP of phylogenetic lineages in clusters of Asian origin only, which may reflect the smaller sample size and reduced power to detect such an association.

The inclusion of *M. africanum* isolates, which have been associated with a lower rate of disease transmission compared to other MTB strains [31], for comparison in our study was not possible due to the very small number of patients infected with this strain. A differential representation of lineages amongst the native Dutch (who are not BCG vaccinated or previously exposed) versus the foreign-born population also represents a possible source of bias. In this dataset, the percentage of lineages circulating in the native Dutch were 7.6%, 10.4%, 25.3% and 36.7% in the CAS, EAI, Beijing and Euro-American lineages, respectively. It should also be noted that the weights used to calculate each patient's propensity to propagate (PPP) in this study depended on the clustering status given by molecular epidemiology data (RFLP- and VNTR-typing) alone, whose accuracy is limited.

In sum, this study demonstrates the importance of controlling for host-related factors in measuring the transmissibility of strains and describes a method to do so in order to identify bacterial factors in future studies. It also shows that there are no significant differences in the ability to propagate of four main phylogenetic lineages in the Netherlands, which is indicative that the spread of imported strains (most often of the EAI, CAS and Beijing lineages) is not necessarily curbed by a lack of adaptation to the native host population.

## Supporting Information

Table S1 **Classification of MIRU and spoligotypes into four lineage groups.**
(DOC)
