# Supplementary figures and images for: A Novel Approach - The Propensity to Propagate (PTP) Method for Controlling for Host Factors in Studying the Transmission of Mycobacterium Tuberculosis
Source: PLoS One. 2014 May 21;9(5):e97816. doi: 10.1371/journal.pone.0097816 (PMC4029888; doi:10.1371/journal.pone.0097816)

**Table S1: Classification of MIRU and spoligotypes into four lineage groups**


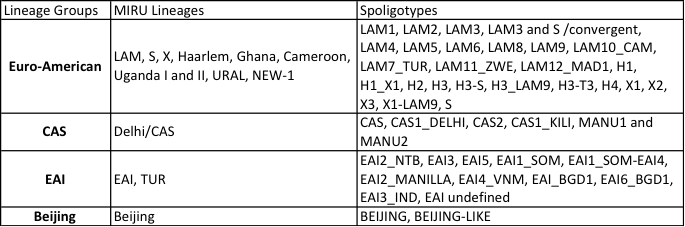

Supplement: Table S1 — Classification of MIRU and spoligotypes into four lineage groups. (DOC) [file pone.0097816.s001.doc]
